# Amnestic Mild Cognitive Impairment Does Not Alter Cerebrocortical Oxygenation Dynamics During Acute Hypoxia–Reoxygenation in Older Adults

**DOI:** 10.3390/biomedicines13112661

**Published:** 2025-10-30

**Authors:** Christopher Cortez, Jewelia Rattanavong, Hannah Dyson, Sarah Ross, Robert T. Mallet, Xiangrong Shi

**Affiliations:** 1 Departments of Pharmacology and Neuroscience, University of North Texas Health Science Center, Fort Worth, TX 76107, USA; christopher.cortez@unthsc.edu (C.C.);; 2 Departments of Internal Medicine, University of North Texas Health Science Center, Fort Worth, TX 76107, USA; 3 Departments of Physiology and Anatomy, University of North Texas Health Science Center, Fort Worth, TX 76107, USA; robert.mallet@unthsc.edu

**Keywords:** frontal cortex, hypoxemia, mild cognitive impairment, near infrared spectroscopy, oxygen extraction

## Abstract

**Background:** This study examined the impact of amnestic mild cognitive impairment (aMCI) on dynamic changes in cerebrocortical oxygen saturation (ScO_2_) and O_2_ extraction during acute, moderately intense, normobaric hypoxia and reoxygenation in elderly adults (71 ± 6 years old). **Methods:** Thirty-two aMCI and thirty-five control subjects participated. Inspired and expired fractions of O_2_ and CO_2_ (mass spectrometry), arterial O_2_ saturation (SaO_2_) and prefrontal ScO_2_ (near-infrared spectroscopy), heart rate, tidal volume and breathing frequency were monitored while subjects breathed hypoxic air (fractional inspired O_2_ 0.10) for 3–5 min (aMCI: 4.5 ± 0.7 min; control: 4.5 ± 0.6 min) and recovered on room air. Values at the pre-hypoxia baseline, the first and last min of hypoxia and the first min of recovery were compared within and between groups using two-factor ANOVA. **Results:** Despite a similar baseline SaO_2_ in aMCI (97.2 ± 1.6%) and control (97.3 ± 1.3%) subjects, prefrontal ScO_2_ was lower (*p* < 0.05) in the aMCI subjects in both the left (67.0 ± 1.7% vs. 69.6 ± 4.5%) and right (66.8 ± 4.6% vs. 69.4 ± 4.1%) hemispheres. Hypoxia similarly decreased SaO_2_ and ScO_2_ in both groups (last min hypoxia, aMCI vs. control subjects: SaO_2_ 76.6 ± 5.3% vs. 77.4 ± 6.1%, left prefrontal ScO_2_ 54.0 ± 4.9% vs. 55.2 ± 6.4%, right prefrontal ScO_2_ 56.0 ± 4.3% vs. 58.2 ± 4.4%). Upon the resumption of room-air breathing, ScO_2_ recovered at similar rates in aMCI and control subjects. **Conclusions:** Although it produced a greater deoxygenation in the left vs. the right prefrontal cortex, acute, normobaric, moderate hypoxia was well tolerated by elderly adults, even those with aMCI. Dynamic changes in cerebral oxygenation during hypoxia and recovery were unaltered by aMCI. Brief, moderate hypoxia does not impose more intense cerebrocortical oxygen depletion in elderly adults with aMCI, despite pre-hypoxic cerebrocortical oxygenation below that of their non-MCI counterparts.

## 1. Introduction

Cerebral hemodynamic responses to acute hypoxia vs. reoxygenation proceed at different rates. Poulin et al. [1] demonstrated that the blood flow velocity of the middle cerebral artery (V_MCA_) monitored using a transcranial Doppler increased less rapidly when normobaric hypoxia was applied to the lower end-tidal partial pressure of O_2_ (P_ET_O_2_) from 100 to 50 mmHg than it fell upon the resumption of room-air breathing. Using arterial spin labeling magnetic resonance spectroscopy, Harris et al. [2] confirmed that blood flows to gray matter changed more gradually and to a lesser extent during the room-air-to-hypoxia transition, when the mean arterial oxygen saturation (SaO_2_) stabilized at ~83% (with P_ET_O_2_ at 52 mmHg) for 20 min, than during the reverse process. Since increases in cerebral blood flow during hypoxic exposures are mediated by the cerebral vasodilatory response to hypoxemia and cerebral tissue hypoxia [3,4,5,6,7], we postulated that the dynamic responses of cerebral tissue oxygenation, like those of cerebral blood flow, would proceed at different rates at the onset of acute hypoxic exposure vs. recovery on room air. However, data comparing the dynamic responses of cerebral tissue oxygenation (ScO_2_) during on-and-off phases of hypoxia are scant. Accordingly, this study examined prefrontal cortical ScO_2_ responses during acute hypoxia vs. room-air reoxygenation.

Mounting evidence suggests that cerebral blood flow is reduced in humans with mild cognitive impairments, i.e., MCI [8,9,10]. Furthermore, Tarumi et al. reported [11] that prefrontal cortical ScO_2_ was lower in subjects with MCI than their cognitively normal counterparts. Murayama et al. [12] examined 113 older adults with cognitive functions ranging from normal to severe impairment and found a direct correlation between mini-mental state examination (MMSE) scores and prefrontal ScO_2_. In our recent report, ScO_2_ responses to either mental or physical challenges were similar in older adults with vs. without MCI, although the baseline ScO_2_ tended to be lower in the MCI subjects [13]. However, whether MCI might affect ScO_2_ responses differently during de- vs. reoxygenation is not known.

Normobaric hypoxic exposures have been proposed as an intervention to slow or prevent cognitive decline in elderly adults [14,15]. Studies in rodents have identified several mechanisms whereby hypoxia may benefit cognitive functions. Hypoxia induced hippocampal neurogenesis in adult rats modeling depression [16] and elicited the cerebrocortical expression of erythropoietin and brain-derived neurotrophic factor in transgenic mice modeling Alzheimer’s disease [17]. Hypoxia also suppressed hippocampal amyloid β (Aβ) plaque formation and pro-inflammatory cytokine expression in transgenic mice and streptozotocin-injected rats modeling Alzheimer’s disease [18,19]. The clinical translation of these studies requires knowledge of the acute effects of hypoxia and reoxygenation on cerebral tissue oxygenation and cardiopulmonary responses in older adults with vs. without cognitive impairments. Accordingly, this study compared ScO_2_ response rates at the onsets of acute hypoxia and recovery in older adults with vs. without amnestic MCI (aMCI).

This study tested the hypothesis that breathing moderately hypoxic gas (10% O_2_) does not produce more severe cerebrocortical O_2_ depletion in elderly adults with aMCI vs. their cognitively normal counterparts, despite the lower cerebrocortical oxygenation before hypoxia in the aMCI subjects. SaO_2_ and bilateral ScO_2_ in the prefrontal cortex were monitored concurrently with inspired and expired O_2_ and CO_2_ fractions and minute ventilation, while the subjects breathed 10% O_2_ for up to 5 min of acute normobaric hypoxia and then recovered while breathing room air. We also hypothesized that the decline in ScO_2_ at the onset of hypoxia is more gradual than ScO_2_ recovery following the resumption of room-air breathing, both in elderly adults with aMCI and cognitively normal elderly subjects.

## 2. Materials and Methods

### 2.1. Study Participants

A total of 32 subjects with aMCI (24 women) and 35 non-MCI control subjects (28 women) were recruited from the Fort Worth area and signed informed consent to participate in the study. The study protocol was reviewed and approved by the North Texas Regional Institutional Review Board for Protection of Human Subjects. After physical screening and an orientation visit to the laboratory, all participants underwent a clinical dementia rating (CDR) evaluation and a battery of neurocognitive function tests including verbal memory and visuospatial memory tests conducted by a geriatric neuropsychologist or psychiatrist in the Geriatric Center at UNT Health Science Center [20]. Amnestic MCI was assessed from the consensus criteria [21,22]. Briefly, subjects were considered to have aMCI if they had a self- or family member-reported memory complaint, a clinical dementia rating ~0.5 and at least one memory testing score more than one standard deviation below the age- and education-adjusted normal group mean, but with normal daily living functionality and no dementia. Neither age nor educational attainment differed between the groups, and the groups had similar medication histories (Table 1).

### 2.2. Study Protocol

During the pre-testing orientation, the testing procedures and instrumentation were explained to and experienced by all subjects. Subjects breathed through air-cushioned disposable facemasks (VacuMed, Ventura, CA, USA) which were fitted to cover the nose and mouth and connected through a Universal Ventilation Meter (UVM; VacuMed, Ventura, CA, USA) to a one-way Hans Rudolph T-tube (Model 2100 VacuMed, Ventura, CA, USA). The inlet of the T-tube was connected to a three-way valve (VacuMed, Ventura, CA, USA), which controlled inflow from the room air or a balloon containing 10% O_2_ (balance N_2_). Subjects who could not tolerate the facemask or had cardiac rhythm abnormalities during orientation were released from the study. During the experiment, subjects breathed poikilocapnic, normobaric hypoxic gas for 5 min or until SaO_2_ fell below 70% for ≥5 s and/or heart rate (HR) increased by ≥30 beats/min—whichever came first. The lab was maintained at 735–745 mmHg barometric pressure, 50–58% relative humidity and 23 ± 1 °C room temperature.

### 2.3. Measurements

During the test, breath-by-breath inspired and expired fractions of O_2_ and CO_2_, collected through a capillary embedded in the proximal inlet of the UVM were analyzed continuously using a mass spectrometer (Perkin-Elmer, 1100 Medical Gas Analyzer, St. Louis, MO, USA) with minimum delay (~0.1 s). Analog signals of breath-by-breath inspiratory tidal volume (TV) and breathing frequency (*f*_Br_) monitored by the UVM were recorded. The UVM was calibrated using a 3-liter calibration syringe (VacuMed, Ventura, CA, USA) for flow rate and the mass spectrometer was calibrated against a medical gas containing 5% CO_2_ and 20% O_2_ (balance N_2_). Heart rate was continuously monitored using three-lead electrocardiography (BIOPAC Model ECG100C, Santa Barbara, CA, USA). Double finger cuffs placed on the proximal phalanges of the index and middle fingers of the non-dominant hand (CNAP 500, Graz, Austria) detected beat-to-beat systolic (SP) and diastolic (DP) arterial pressures. Mean arterial pressure (MAP) was computed off-line as one-third of SP plus two-thirds of DP.

Systemic arterial O_2_ saturation (SaO_2_) was monitored in the right earlobe by a transcutaneous sensor (TOSCA 500, Radiometer America Inc., Westlake, OH, USA) maintained at 42 °C to dilate and thereby arterialize the cutaneous capillary blood in the earlobe. Cerebral tissue O_2_ saturation (ScO_2_) of the prefrontal cortex was monitored using NIRS (NIRO-200, Hamamatsu Photonics, Bridgewater, NJ, USA) with a pair of sensors placed on the subject’s forehead at an analog signal output of 1 Hz. All measurements were carried out with the subject in supine position. Analog data were continuously digitized on-line at 250 Hz by a computer interfaced with a data acquisition system (MP150 BIOPAC, Santa Barbara, CA, USA).

**Figure 1:**
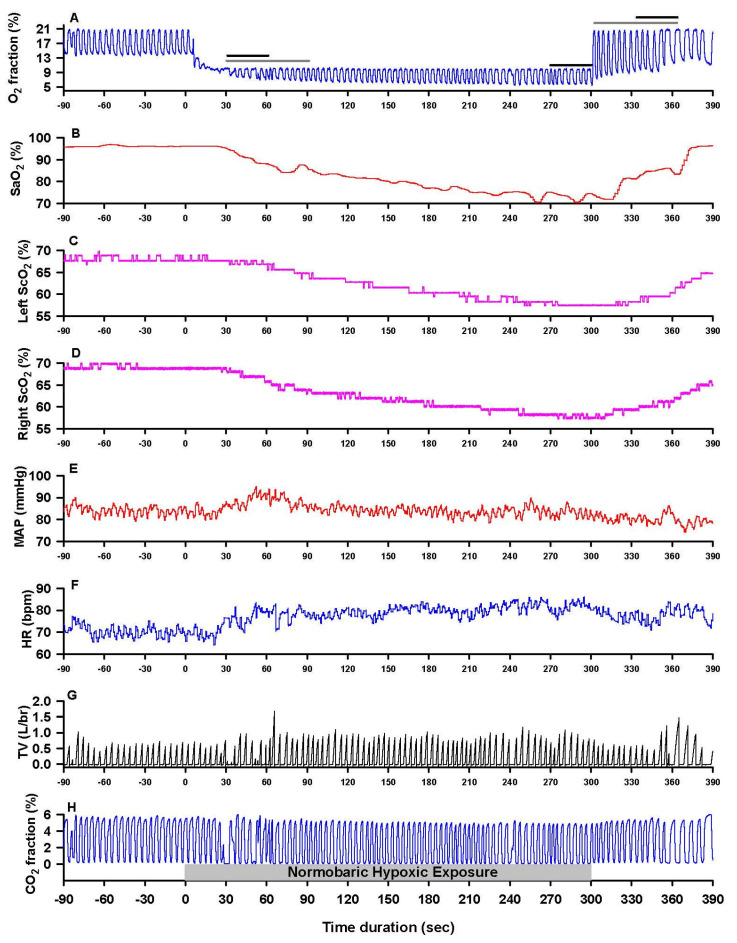
Hypoxia–reoxygenation protocol: monitored variables. (**A**) inspired (peak of cycle) and expired (nadir of cycle) O_2_ fractions; (**B**) arterial oxygen saturation (SaO_2_); left (**C**) and right (**D**) prefrontal cortex oxygenation (ScO_2_); (**E**) mean arterial pressure (MAP); (**F**) heart rate (HR); (**G**) inspiratory tidal volume (TV); (**H**) inspired and expired fractions of CO_2_. Pre-hypoxia baseline is from −90 s to 0 s, followed by 5 min normobaric hypoxia (fractional inspired O_2_ 0.10) and then 90 s recovery breathing room air. Baseline values are the mean values from c. 60 s of continuous data acquisition before the hypoxic exposure. Values for the first and final min of hypoxia and the first min of recovery were taken as the mean values from 30 to 60 s of each interval (indicated by horizontal black lines in panel **A**). The horizontal black lines (panel **A**) indicate the data averaged for the first and last minutes of hypoxic exposure and the first minute of recovery, respectively. The horizontal gray lines (panel **A**) indicate the initial deoxygenation phase of hypoxic breathing (equilibration of the inspired air inside facemask takes ~30 s) and the reoxygenation phase during the first min of recovery, respectively. Data are from a 71-year-old female subject.

After instrumentation and ≥20 min supine relaxation, baseline values were taken, and then hypoxic ventilation (10% O_2_) was initiated. The duration of hypoxic ventilation did not differ (*p* = 0.825) in the aMCI (273 ± 43 s; 180–300 s) vs. control subjects (271 ± 37 s; 185–300 s). Figure 1 presents a typical recording of the monitored variables during the experiment.

### 2.4. Data Analysis and Statistics

A ~60 s segment of continuous, stable analog data collected before hypoxic exposure was averaged to obtain baseline values (designated Base). Data collected at 30–60 s of the first and last minutes of hypoxic exposure (designated H-1 and H-2) and the first min of recovery (designated Rec) were averaged to represent the values at the respective experimental stages (see Figure 1), as in previous studies [23,24,25]. Additional 60 s segments were extracted after the initial ~30 s of breathing 10% O_2_ gas, i.e., after equilibration of hypoxic gas in the facemask, and during the 1st min of room-air breathing after hypoxia (Figure 1). Data from consecutive 10 s intervals of these segments were averaged, and the slopes of the least-squares regressions were computed, yielding the de- and reoxygenation response rates. Partial pressures of end-tidal O_2_ (P_ET_O_2_) and CO_2_ (P_ET_CO_2_) were estimated from expired fractions of O_2_ and CO_2_ multiplied by the ambient barometric pressure. Left and right prefrontal cortical oxygen extraction fractions (O_2_EF) equaled (SaO_2_–ScO_2_)/SaO_2_.

Frequencies of categorical variables in the aMCI and control groups were compared with Fisher’s exact test for two-tailed probability. Scalar variables of basic characteristics in the two groups (Table 1) were compared using Student’s *t*-test for two independent groups. Group data at different timepoints were analyzed with two-factor ANOVA to assess the impacts of group and time factors. ScO_2_ slopes (i.e., ΔScO_2_/s) during de- and reoxygenation were calculated from least-square linear regression. Three-factor ANOVA was applied to compare ΔScO_2_/s slopes in aMCI vs. control groups, de- vs. reoxygenation phases and left vs. right cerebral hemispheres during the first 60 s of hypoxia and room-air recovery. Post hoc analysis with Tukey’s Studentized Range test was applied to factors identified as statistically significant through ANOVA.

Numerical values are reported as group means ± standard deviation (SD) of the means. Pearson correlation coefficients were determined for the associations between ScO_2_ and O_2_EF, and a general linear model procedure was applied for the interaction of group and cerebral hemisphere factors on the association. Statistical significance was accepted at *p*-values ≤ 0.05. Statistical analyses were performed with Statistical Analysis System software (SAS Version 9.4, Cary, NC, USA).

## 3. Results

### 3.1. Cardiopulmonary Responses to Hypoxia and Reoxygenation

During hypoxia, the MAP tended to decrease (time factor *p* = 0.033), while the HR increased as expected (time factor *p* < 0.0001). At 60 s of recovery, neither the MAP nor the HR differed significantly from the respective baselines. Ventilatory responses to hypoxia and reoxygenation were similar in the aMCI and control subjects. The tidal volume trended upward (time factor *p* = 0.037), and breathing frequency increased marginally (time factor *p* = 0.055) during hypoxia, while minute ventilation increased as expected (time factor *p* < 0.0001), producing hypocapnia, indicated by a reduced P_ET_CO_2_ (time factor *p* < 0.0001). Although breathing frequency during hypoxia trended higher in the aMCI vs. control subjects (group factor *p* = 0.004), the post hoc analysis did not reveal a statistical significance at any timepoint. During the recovery, minute ventilation fell below the baseline in the control subjects (Table 2).

### 3.2. Cerebrocortical Oxygenation During Hypoxia and Reoxygenation

**Figure 2:**
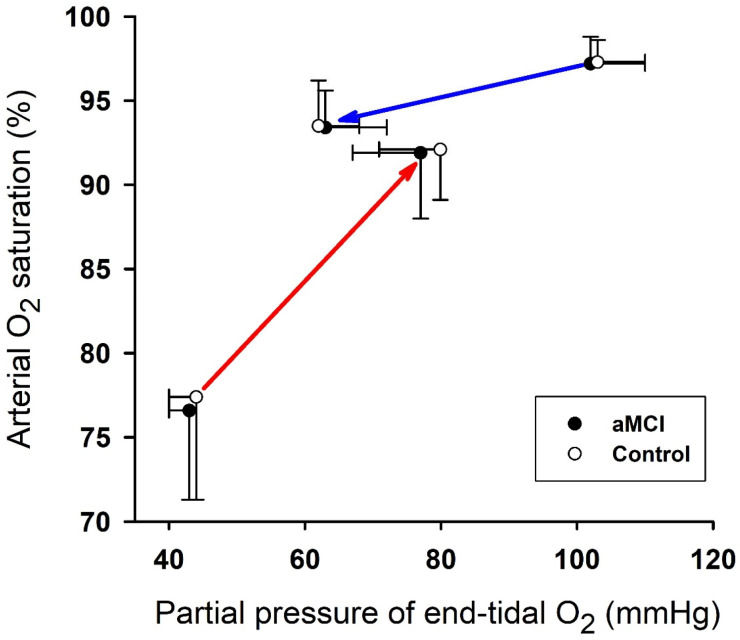
Changes in hemoglobin O_2_ saturation during hypoxia and reoxygenation. Changes in SaO_2_ vs. P_ET_O_2_ from the baseline to the 1st min of hypoxic exposure (blue arrow) and from the last min of hypoxia to the 1st min of recovery (red arrow) demonstrate different rates of hemoglobin-O_2_ disassociation vs. reassociation at the onsets of hypoxia vs. recovery, respectively. Mean values ± SD are from Table 2. Because the aMCI and control values were nearly identical, the overall mean values of the two groups determined the positions of the arrows. The SaO_2_/ΔP_ET_O_2_ slope was greater (*p* < 0.05) during O_2_ reassociation (0.43%/mmHg) than disassociation (0.10%/mmHg).

At the pre-hypoxia baseline, ScO_2_ in both the left and right prefrontal cortices was lower (group factor *p* < 0.001) in the aMCI vs. cognitively normal subjects (Table 2). P_ET_O_2_ and SaO_2_ in both groups fell within the first min of hypoxia, but ScO_2_ did not fall significantly (Table 2). SaO_2_ during the last min hypoxia was appreciably lower (~77%) than the respective baseline and first min hypoxia values in both groups. By the last min of hypoxia, ScO_2_ had fallen significantly below the baseline in both groups, and to a greater extent in the left vs. right prefrontal cortex (hemisphere factor *p* = 0.015). The post hoc analysis revealed a significant difference between the left and right cortical ScO_2_ in the control group during the last min of hypoxia (Table 2). The SaO_2_ recovery upon the resumption of room-air breathing was more rapid than the SaO_2_ decline at the onset of hypoxia (Figure 2). Within 1 min of recovery on room air, SaO_2_ and ScO_2_ in both groups returned to ~92% and ~90% of the respective baseline values, but remained significantly below their respective baselines (Table 2).

Cerebrocortical O_2_EF (Table 2) was significantly affected by group (*p* < 0.0001), time (*p* < 0.0001) and hemisphere (*p* = 0.007) factors. However, only in the right prefrontal cortex did the post hoc analysis reveal a statistically significant difference in O_2_EF between the aMCI and control groups. O_2_EF did not change during the first min of hypoxia in either group, but fell significantly below the baseline by the last min of hypoxia in the right but not the left prefrontal cortex in both aMCI and control subjects. Although prefrontal O_2_EF during the first min of room-air recovery trended upward in both groups, O_2_EF differed significantly from the baseline only in the right prefrontal cortex. O_2_EF fell further in the right vs. left cortex during hypoxia, but the post hoc analysis revealed a statistically significant difference between the hemispheres only in the control group during the last min of hypoxic ventilation (Table 2).

**Figure 3:**
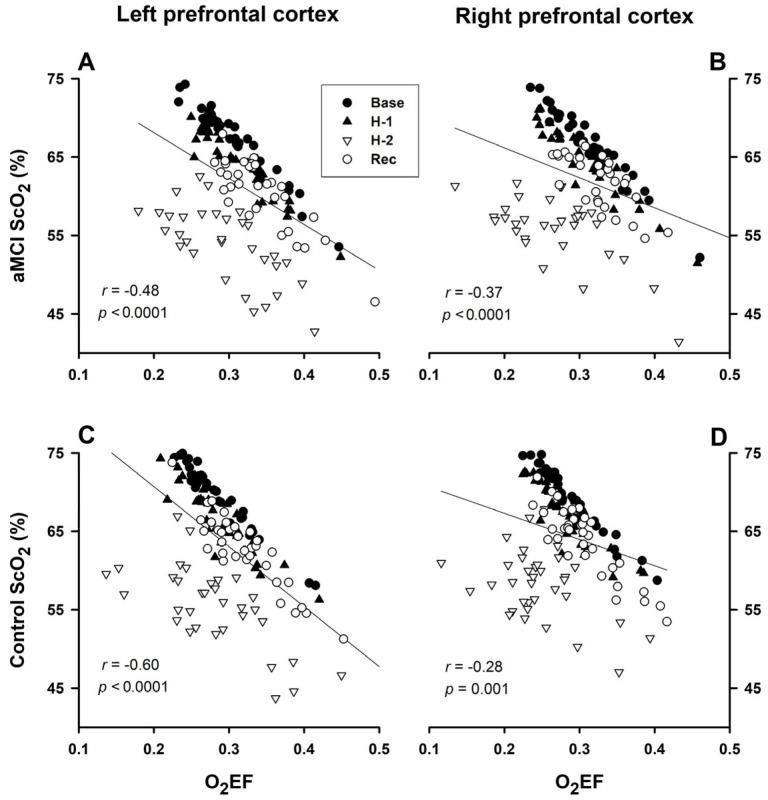
Correlation of ScO_2_ with O_2_EF. Decreases in ScO_2_ significantly correlated with increases in O_2_EF in both aMCI (panels (**A**,**B**)) and control (panels (**C**,**D**)) subjects. The slopes of ScO_2_/O_2_EF (%/fraction) were not influenced by the group × O_2_EF interaction factor in the left (*p* = 0.169) and right (*p* = 0.713) prefrontal cortices. However, the ScO_2_/O_2_EF slope was significantly steeper in the left (−76.5 ± 8.7) than the right (−33.5 ± 9.8) prefrontal cortex in the control group (cerebral hemisphere × O_2_EF interaction factor *p* = 0.002), and not different between the left (−58.5 ± 9.7) vs. right (−38.3 ± 8.6) prefrontal cortex in the aMCI group (cerebral hemisphere × O_2_EF interaction factor *p* = 0.112).

Decreases in ScO_2_ were significantly correlated with increases in O_2_EF in both the left and right prefrontal cortices of the aMCI (Figure 3A,B) and control (Figure 3C,D) subjects. The ΔSaO_2_/ΔO_2_EF slopes (%/fraction) in the left and right prefrontal cortices were −58.5 ± 9.7 and −38.3 ± 8.6 in the aMCI subjects and −76.5 ± 8.7 and −33.5 ± 9.8 in the controls, respectively. The ΔSaO_2_/ΔO_2_EF slopes were significantly steeper in the left vs. right prefrontal cortex in the control group (side × O_2_EF interaction *p* = 0.002), but were not significantly different in the aMCI group (side × O_2_EF interaction *p* = 0.112). These slopes did not differ significantly in the aMCI vs. control groups in the left (group × O_2_EF interaction *p* = 0.169) or right (group × O_2_EF interaction *p* = 0.713) hemispheres.

### 3.3. Rates of De- and Reoxygenation

**Figure 4:**
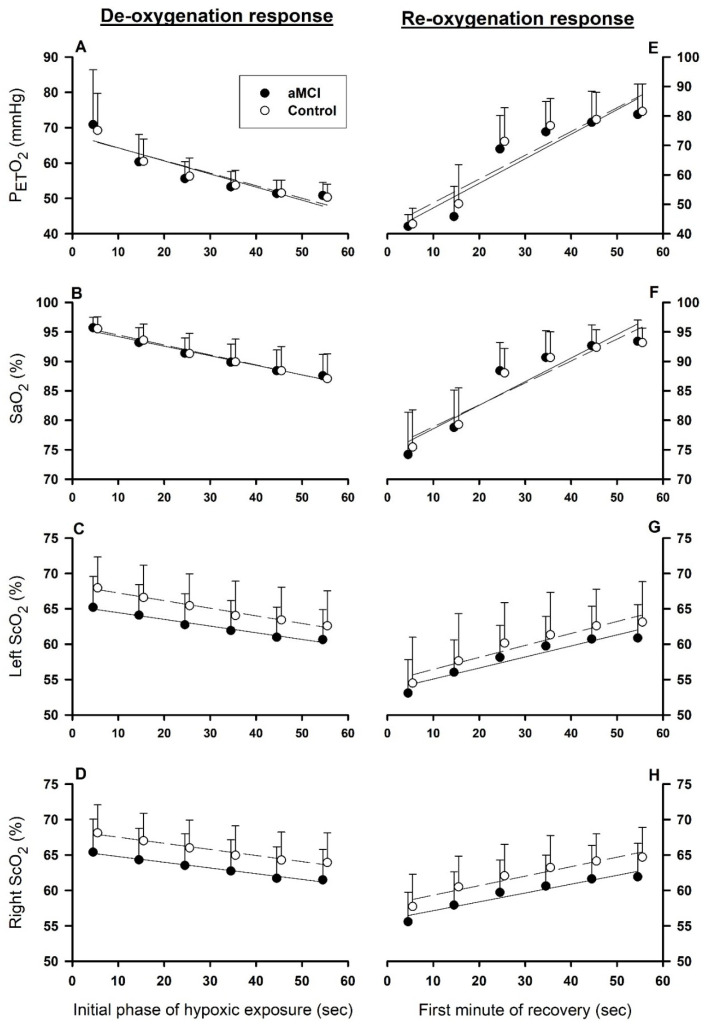
Dynamic responses of arterial and cerebrocortical oxygen saturation during de- and reoxygenation. Left (**A**–**D**) and right (**E**–**H**) panels show the responses of partial pressure of end-tidal O_2_ (P_ET_O_2_), arterial oxygen saturation (SaO_2_) and left and right cerebrocortical oxygen saturation (ScO_2_) at 30–90 s hypoxia (panels **A**–**D**) and first min of reoxygenation (panels **E**–**H**), respectively. Mean values (circles) ± SD.

Figure 4 presents the P_ET_O_2_, SaO_2_ and ScO_2_ group responses during the first 60 s of hypoxia (Figure 4A–D) and the first 60 s of recovery (Figure 4E,F). P_ET_O_2_ closely paralleled the dynamic changes in SaO_2_ in the aMCI and cognitively normal subjects. In both groups, P_ET_O_2_, SaO_2_ and left and right prefrontal ScO_2_ fell progressively during the first min of hypoxia (Figure 4A–D), and recovered over the first 60 s of reoxygenation (Figure 4E–H). Time × group interaction factors revealed no statistically significant between-group differences in the rates of change in these variables during de- or reoxygenation. However, ScO_2_ values in both the left and right prefrontal cortices were consistently lower in the aMCI subjects vs. controls.

None of the slopes of P_ET_O_2_, SaO_2_ and left and right ScO_2_ vs. time (s) differed significantly between the aMCI and control groups during the initial phase of acute hypoxia (deoxygenation phase) and first min of reoxygenation (Table 3). ΔScO_2_/s (Table 3) revealed a more rapid deoxygenation in the left vs. right prefrontal cortex in aMCI and control groups. In both groups, the ΔP_ET_O_2_/s, ΔSaO_2_/s and left and right prefrontal ScO_2_/s slopes were significantly greater (phase factor *p* < 0.0001) during reoxygenation vs. deoxygenation.

**Figure 5:**
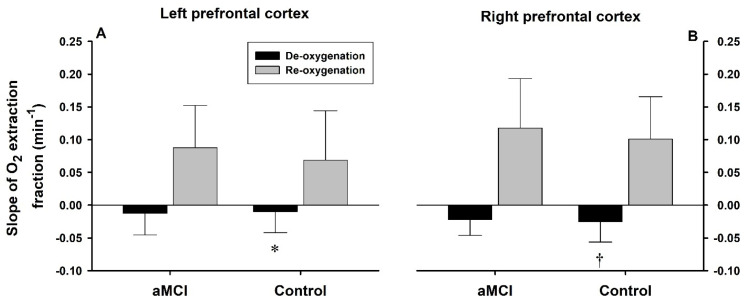
Slopes of oxygen extraction fractions during hypoxia and reoxygenation. Left (**A**) and right (**B**) prefrontal cortical O_2_EF decreased with time during initial phase of deoxygenation, breathing hypoxic air, and increased with time during the first min of reoxygenation, breathing room air during recovery. The decline of O_2_EF (absolute unit) during deoxygenation was substantially slower than the rate of O_2_EF recovery during reoxygenation (phase factor *p* < 0.001). The O_2_EF slopes did not differ between the aMCI and control subjects (group factor *p* = 0.179). Overall, the rate of O_2_EF change was greater in the right than the left prefrontal cortical tissues (side factor *p* = 0.001). *: O_2_EF slope not significantly different from zero (*p* = 0.082); ^†^: *p* < 0.05 vs. left prefrontal cortex in the same group during deoxygenation phase.

Rates of O_2_EF changes (Figure 5) were similar in the aMCI and control groups (group factor *p* = 0.179). The ΔO_2_EF/min slopes in the left and right prefrontal cortices, respectively, were −0.012 ± 0.033 and −0.022 ± 0.024 in the aMCI subjects vs. −0.010 ± 0.032 and −0.025 ± 0.031 in the controls during deoxygenation, and 0.088 ± 0.064 and 0.118 ± 0.085 in the aMCI group vs. 0.069 ± 0.075 and 0.101 ± 0.065 in the controls during recovery. Both phase (*p* < 0.001) and hemisphere factors (*p* = 0.001) significantly affected the ΔO_2_EF/min slopes. During deoxygenation, the ΔO_2_EF/min slope was steeper in the right vs. left cortex in the control but not the aMCI subjects.

## 4. Discussion

This study demonstrated that the declines of P_ET_O_2_, SaO_2_ and ScO_2_ at the onset of acute, normobaric, moderate (10% O_2_) hypoxia were considerably slower than their respective recoveries upon the resumption of room-air breathing. However, none of these responses differed significantly in elderly adults with aMCI vs. their cognitively normal counterparts. Although prefrontal cortical ScO_2_ was significantly lower in the aMCI than in the cognitively normal subjects before hypoxia despite similar SaO_2_ values, during hypoxia, neither left nor right prefrontal ScO_2_ differed in aMCI vs. control subjects. The lower left and right prefrontal ScO_2_ values in the aMCI vs. cognitively normal subjects are concordant with a previous report [11], and may be related to a lower cerebral perfusion in elderly adults with MCI [8,9,10].

The rates of ScO_2_ changes at the onset of hypoxia and upon the resumption of room-air breathing were anatomically asymmetric, with more rapid responses in the left vs. the right prefrontal cortex. Furthermore, during hypoxia, ScO_2_ fell to a greater extent in the left vs. the right prefrontal cortex in the cognitively normal subjects, but not the aMCI subjects. The greater stability of right vs. left prefrontal ScO_2_ was associated with decreased O_2_EF during hypoxia; consequently, a significant inverse correlation emerged between ScO_2_ and O_2_EF in the right prefrontal cortex.

### 4.1. Hypoxemia and Cerebral Tissue Hypoxia During Hypoxia vs. Reoxygenation

The SaO_2_ decline at the onset of hypoxia was considerably slower than the SaO_2_ recovery upon the resumption of room-air breathing, and P_ET_O_2_ closely paralleled the dynamic changes in SaO_2_ in the aMCI and cognitively normal subjects. The mechanisms underlying the different SaO_2_ response rates are unclear. We propose that the different rates may reflect the nonlinear changes in the O_2_ affinity of hemoglobin as arterial PO_2_ changes during the baseline-to-hypoxia and hypoxia-to-reoxygenation transitions (Figure 2). Due to hemoglobin’s high O_2_ saturation at PO_2_ >60 mmHg, SaO_2_ would decline only gradually until arterial PO_2_ reaches the “shoulder” of the sigmoid hemoglobin O_2_ saturation curve (Figure 2: red arrow). Conversely, the resumption of room-air breathing would produce rapid increases in SaO_2_ as arterial PO_2_ recovered from its nadir of *c*. 40 mm Hg, where the O_2_ saturation curve is steep (Figure 2: blue arrow).

Paralleling SaO_2_, ScO_2_ changes in both prefrontal cortices were more gradual during de- than reoxygenation in both the aMCI and control subjects. The slower ScO_2_ decline at hypoxia onset vs. its recovery upon reoxygenation may contribute to the previously reported slower changes in cerebral blood flow during hypoxic vs. normoxic respiration [2,5]. These data are in accordance with the cerebrovascular vasodilatory response to maintain cerebral O_2_ delivery during hypoxemia. Indeed, smaller cerebral hyperemic responses during hypoxia were associated with smaller ScO_2_ responses [2,5]. On the other hand, ScO_2_ recovery and, thus, cerebrocortical O_2_ replenishment during reoxygenation were more rapid than the antecedent declines during hypoxia. Furthermore, the robust ScO_2_ response to reoxygenation was associated with a rapid increase in cerebrocortical O_2_EF (Figure 4) in the left and right prefrontal cortices, helping “repay” the cerebrocortical O_2_ debt accrued during hypoxia in elderly adults with or without aMCI.

### 4.2. Impact of MCI on Cerebral O_2_ Dynamics During Hypoxia–Reoxygenation

Cerebrocortical O_2_ saturation was reportedly higher in the right vs. the left prefrontal cerebral cortex in right-handed young adults [26]. However, Olopade et al. [27] reported no difference between left and right ScO_2_ in young healthy adults and those with sleep apnea, although overall ScO_2_ was significantly lower in the sleep apnea subjects. The present study demonstrated a significantly lower ScO_2_ at baseline in both prefrontal cortices in the aMCI subjects vs. their cognitively normal counterparts, although the baseline ScO_2_ did not differ in the right vs. left cortex in either group (Table 2). Nonetheless, ScO_2_ tended to change more rapidly in the left vs. the right prefrontal cortex during both de- and reoxygenation in both elderly groups (Table 3). Moreover, ScO_2_ during the last min of hypoxia was appreciably lower in the left vs. the right prefrontal cortex in the control group (Table 2). This observation aligns with Ide’s study in young adults, where hypoxic exposure with P_ET_O_2_ clamped at 45 mmHg for 10 min elicited a greater ScO_2_ reduction in the left than the right prefrontal cortex [28]. A greater ScO_2_ reduction during hypoxia could be ascribed to a less robust increase in cerebral blood flow and/or greater tissue oxygen consumption in the left vs. the right cortex. Since increases in total cortical hemoglobin content (an index of perfusion) during hypoxic exposure did not differ in the left vs. the right cortex [28], the greater ScO_2_ reduction in the left prefrontal cortex is more likely related to a greater tissue oxygen consumption or metabolic demand/rate than the contralateral cortex, as indicated by the greater O_2_EF in the left vs. the right cortex (Table 2 and Figure 3).

Interestingly, the differences in ScO_2_ and O_2_EF of left vs. right prefrontal cortices were more appreciable in the cognitively normal subjects than their aMCI counterparts (Table 2, Figure 3), suggesting that the cerebral metabolic demand/rate was greater in the left than the right hemisphere, and this cerebrocortical asymmetry might be diminished with aMCI. Although the mechanism for the more rapid ScO_2_ recovery in the left vs. the right prefrontal cortex was not clear, it may represent a repayment of the greater O_2_ debt accrued during the antecedent hypoxia in the left prefrontal cortex, which experienced the greater hypoxic decrease in ScO_2_.

### 4.3. Study Limitations and Perspectives

Cerebral tissue oxygenation represents the dynamic balance between O_2_ delivery, i.e., cerebral blood flow times arterial O_2_ content, and tissue O_2_ utilization for oxidative metabolism; consequently, cerebral blood flow is a central determinant of cerebral tissue oxygenation. Cerebral blood flow, which in human subjects typically is estimated from middle cerebral artery flow velocities monitored using transcranial Doppler sonography [23], was not assessed in this study, due to the technical challenges of bilateral transcranial Doppler instrumentation in subjects wearing facemasks and bilateral sensors for near-infrared spectroscopy. The anterior cerebral artery perfuses the superior and lateral portions of the prefrontal cortex [29]; consequently, the flow velocity in the middle cerebral artery might not represent the region where ScO_2_ was monitored. The ScO_2_ responses in the two cerebral hemispheres differed unexpectedly, yet we could not discern if the greater ScO_2_ reduction in the left prefrontal cortex during hypoxia was ascribable to a less robust cerebral blood flow response and/or greater O_2_ extraction in the left vs. the right prefrontal cortex.

The dynamic ScO_2_ response was slower at the onset of hypoxia vs. reoxygenation. Since cerebral tissue O_2_ content regulates cerebral perfusion [30], the different ScO_2_ response rates could explain the previously reported [1,2] slower cerebral blood flow responses to acute hypoxia vs. recovery on room air.

The extent to which short-term memory is affected by low pre-hypoxia ScO_2_ in the aMCI group and whether chronic cerebral under-perfusion or hypoxia may induce adaptive angiogenesis [31] to minimize or prevent low ScO_2_ in aMCI are as of yet unknown. Such adaptations may alter the trajectory of neurodegenerative progression from aMCI to Alzheimer’s disease and/or dementia. Indeed, repeated bouts of hypoxia, similar in intensity and duration to the hypoxia exposures of this study, are proposed to slow or interrupt cognitive decline in older adults [15]. Studies in rodent models of cognitive impairment identified potential mechanisms underlying the cognitive benefits of intermittent hypoxia. Hypoxia promoted hippocampal neurogenesis [16] and suppressed amyloid β (Aβ) plaque formation and pro-inflammatory tumor necrosis factor α expression [19] in rat models of cognitive impairment. In transgenic mice modeling Alzheimer’s disease, intermittent hypoxia activated cerebrocortical erythropoietin and brain-derived neurotrophic factor expression [17], and attenuated Aβ plaque formation and the expression of pro-inflammatory interleukins 1 and 6 [18]. Whether cyclic hypoxia of the duration and intensity of the present exposures elicits such adaptations in elderly humans, and to similar extents in aMCI vs. cognitively intact adults, is unclear, although the cerebrocortical deoxygenation during hypoxia was similar in the aMCI and control groups. Although medication use was similar in the aMCI and control subjects, the potential impact of medications on the studied responses cannot be excluded.

### 4.4. Conclusions

This study is the first to demonstrate that SaO_2_ and ScO_2_ change more gradually at the onset of hypoxic breathing than reoxygenation in elderly adults. However, the rates of these dynamic SaO_2_ and ScO_2_ responses were similar in elderly adults with aMCI vs. their cognitively normal counterparts. ScO_2_ declines during hypoxia and recovers upon reoxygenation more rapidly in the left vs. the right prefrontal cortex, both in aMCI and cognitively normal elderly adults. Although pre-hypoxia ScO_2_ was lower in both hemispheres in the aMCI vs. cognitively normal subjects despite similar SaO_2_ values, these ScO_2_ differences subsided during hypoxia. Thus, brief exposures to moderate, normobaric hypoxia do not impose more intense cerebrocortical hypoxia in elderly adults with aMCI vs. their cognitively normal counterparts.

## Figures and Tables

**Table 1:** Characteristics, neurobehavioral test scores and medications of aMCI and control subjects.

	aMCI (*n* = 32)	Control (*n* = 35)	*t*	*p*
Men, Women	8, 24	7, 28	−	0.771
Age (year)	71.1 ± 5.7	71.2 ± 5.8	−0.06	0.955
Weight (kg)	72.8 ± 17.1	76.4 ± 14.4	−0.92	0.361
Height (m)	1.66 ± 0.09	1.67 ± 0.10	−0.58	0.568
Education (year)	16.2 ± 1.3	15.9 ± 1.8	0.86	0.396
Geriatric Depression Scale (score)	1.1 ± 1.2	1.0 ± 1.2	0.22	0.830
Clinical Dementia Rating (point)	0.48 ± 0.09	0.33 ± 0.24	3.57	0.001
Mini-mental State Examination (point)	27.7 ± 1.4	28.7 ± 1.1	−3.16	0.003
Trail Making Test–version A (sec)	44 ± 17	29 ± 7	4.67	<0.001
Trail Making Test–version B (sec)	128 ± 61	71 ± 18	5.05	<0.001
Verbal Memory (word)	5.8 ± 1.9	7.6 ± 1.8	−3.65	0.001
Visuospatial Memory (point)	5.3 ± 2.6	8.6 ± 1.8	−5.91	<0.001
Right vs. Left Handedness	27 vs. 5	30 vs. 5	−	1.000
Duration of Hypoxic Exposure (sec)	273 ± 43	271 ± 37	0.22	0.825
***Number (%) of subjects with prescribed medications***				
**Category of Medication**	**aMCI**	**Control**	**Total**	***p***
Hypertension/Coronary Arterial Disease	17 (53.1)	12 (34.3)	29 (43.3)	0.309
Hyper-cholesterol/Hyper-lipidemia	12 (37.5)	11 (31.4)	23 (34.3)	0.618
Hyperglycemia	7 (21.9)	3 (8.6)	10 (14.9)	0.175
Anxiety/Depression	15 (46.9)	9 (25.7)	24 (36.8)	0.081
Reflux/Gastric Acid	9 (28.1)	10 (28.6)	19 (28.4)	1.000
Hypothyroid/Hyperthyroid	6 (18.8)	11 (31.4)	17 (25.4)	0.384
Allergy	9 (28.1)	8 (22.9)	17 (25.4)	0.780
Sleep Aid	7 (21.9)	3 (8.6)	10 (14.9)	0.175
Hormone Replacement	6 (18.8)	4 (11.4)	10 (14.9)	0.501

Scalar values are group means ± standard deviation. *p* values are from comparisons of scalar variables in the aMCI vs. control subjects using *t*-test for two independent groups, and from comparisons of categorical subject distributions in the two groups using Fisher’s exact test for two-tailed probability. *t*: unpaired *t* test statistic; *p*: probability the null hypothesis is true. Verbal memory and visuospatial memory: 10 min delayed free recall of the words and 30 min delayed recall of the sketch shape and location, respectively.

**Table 2:** Minute data during the experiment.

	aMCI Subjects				Control Subjects				ANOVA		
	Base	H-1	H-2	Rec	Base	H-1	H-2	Rec	Factor	F	*p*
HR (bpm)	67 ± 10	70 ± 10	77 ± 11 *	71 ± 10	69 ± 10	71 ± 11	78 ± 11 *	72 ± 10	Group Time	0.83 9.26	0.3636 <0.0001
MAP (mmHg)	95 ± 10	97 ± 9	94 ± 10	92 ± 9	96 ± 9	96 ± 10	93 ± 10	91 ± 10	Group Time	0.29 2.95	0.593 0.033
TV (L)	0.80 ± 0.32	0.88 ± 0.30	0.99 ± 0.45	0.93 ± 0.45	0.88 ± 0.37	1.01 ± 0.40	1.12 ± 0.49	0.92 ± 0.49	Group Time	2.56 2.86	0.1107 0.0374
*f*_Br_ (c/m)	12.6 ± 3.9	13.0 ± 4.5	12.9 ± 5.6	11.2 ± 5.1	11.0 ± 4.4	11.3 ± 4.6	11.5 ± 4.4	9.4 ± 4.0	Group Time	8.42 2.57	0.0040 0.0549
Vent (L/m)	9.5 ± 3.7	10.7 ± 3.1	11.4 ± 4.1	9.3 ± 4.9	8.8 ± 2.4	10.6 ± 3.6	12.0 ± 5.0 *	7.8 ± 3.2 *	Group Time	0.77 8.79	0.3824 <0.0001
P_ET_CO_2_ (mmHg)	43 ± 3	41 ± 4	38 ± 4 ^§^	41 ± 4	43 ± 3	42 ± 3	39 ± 3 ^§^	42 ± 3	Group Time	0.80 19.99	0.3711 <0.0001
P_ET_O_2_ (mmHg)	102 ± 8	63 ± 9 ^§^	43 ± 3 ^§^	77 ± 10 ^§^	103 ± 7	62 ± 6 ^§^	44 ± 4 ^§^	79 ± 9 ^§^	Group Time	0.64 772.9	0.425 <0.0001
SaO_2_ (%)	97.2 ± 1.6	93.4 ± 2.2 *	76.6 ± 5.3 ^§^	91.9 ± 3.9 *	97.3 ± 1.3	93.5 ± 2.7 *	77.4 ± 6.1 ^§^	92.1 ± 3.0 *	Group Time	0.33 399.3	0.564 <0.0001
L-ScO_2_ (%)	67.0 ± 4.7	64.0 ± 4.3	54.0 ± 4.9 ^§^	60.5 ± 4.5 *	69.6 ± 4.5 ^†^	66.6 ± 4.4 ^†^	55.2 ± 6.4 ^§^	62.1 ± 5.7 *	Group Time Side	31.31 175.2 6.01	<0.0001 <0.0001 0.015
R-ScO_2_ (%)	66.8 ± 4.6	64.4 ± 4.5	56.0 ± 4.3 ^§^	61.4 ± 4.5 *	69.4 ± 4.1 ^†^	67.0 ± 3.9 ^†^	58.2 ± 4.4 ^§†‡^	63.8 ± 4.5 *^†^			
L-O_2_EF	0.31 ± 0.05	0.31 ± 0.05	0.29 ± 0.06	0.34 ± 0.05	0.28 ± 0.05	0.29 ± 0.04	0.29 ± 0.08	0.33 ± 0.06	Group Time Side	24.90 22.90 7.56	<0.0001 <0.0001 0.007
R-O_2_EF	0.31 ± 0.05	0.31 ± 0.05	0.27 ± 0.06 *	0.33 ± 0.05 ^§^	0.29 ± 0.04 ^†^	0.28 ± 0.04 ^†^	0.25 ± 0.05 *^‡^	0.31 ± 0.05 ^§^			

Base: baseline; H-1 and H-2: first and last min hypoxic exposure, respectively; Rec: first min recovery on room air; F: F test statistic; *p*: probability the null hypothesis is true. HR: heart rate; MAP: mean arterial pressure; TV: inspiratory tidal volume; *f*_Br_: breathing frequency; Vent: minute ventilation; P_ET_CO_2_ and P_ET_O_2_: partial pressures of end-tidal CO_2_ and O_2_, respectively; SaO_2_: arterial oxygen saturation; L-ScO_2_ and R-ScO_2_: O_2_ saturation of left and right prefrontal cortices, respectively; L-O_2_EF and R-O_2_EF: O_2_ extract fraction of left and right prefrontal cortices, respectively. F: F value from ANOVA determined by the ratio of the variance between the group means to the variance within the groups. * *p* < 0.05 vs. baseline of the same group; ^§^ *p* < 0.05 vs. all other timepoints within the group; ^†^ *p* < 0.05 vs. aMCI; ^‡^ *p* < 0.05 vs. L-ScO_2_. There were no significant interactions of group × time factors for any of these variables. *n* = 32 aMCI and 35 normal subjects, respectively, except for P_ET_O_2_ (29 aMCI subjects, 32 control subjects).

**Table 3:** Response rates of P_ET_O_2_, SaO_2_ and left and right prefrontal ScO_2_ during initial phase of acute hypoxia exposure and first min of recovery.

	aMCI (*n* = 32)		Control (*n* = 35)		ANOVA		
	Hypoxia	Recovery	Hypoxia	Recovery	Factor	F	*p*
P_ET_O_2_/time (mmHg/s)	−0.37 ± 0.22	0.83 ± 0.21 *	−0.35 ± 0.19	0.81 ± 0.21 *	Group Phase	0.33 157.85	0.568 <0.0001
SaO_2_/time (%/s)	−0.16 ± 0.06	0.40 ± 0.13 *	−0.17 ± 0.05	0.37 ± 0.12 *	Group Phase	0.38 179.10	0.539 <0.0001
L-ScO_2_/time (%/s)	−0.09 ± 0.04	0.16 ± 0.07 *	−0.11 ± 0.04	0.17 ± 0.06 *	Group Phase Hemisphere	2.80 75.94 17.42	0.096 <0.0001 <0.0001
R-ScO_2_/time (%/s)	−0.08 ± 0.03	0.12 ± 0.05 *^‡^	−0.09 ± 0.03 ^‡^	0.13 ± 0.06 *^‡^			

P_ET_O_2_: partial pressure of end-tidal O_2_; SaO_2_: arterial oxygen saturation; L-ScO_2_ and R-ScO_2_: oxygenation in left and right prefrontal cortices, respectively. *: *p* < 0.05, hypoxia vs. recovery; ^‡^: *p* < 0.05, left vs. right cortex in the same phase and group (2- or 3-factor ANOVA). Values are group means ± standard deviation of the means.

## Data Availability

The datasets used and/or analyzed during the current study are available from the corresponding author upon request.
